# Metabolic syndrome among Tunisian psychiatric patients receiving antipsychotics: Sex based comparison

**DOI:** 10.1192/j.eurpsy.2026.11788

**Published:** 2026-07-31

**Authors:** Y. Ben Othmene, A. Aissa, A. Helaoui, M. Gardabou, R. Hosni, A. Ouertani, R. Jomli

**Affiliations:** 1 Avicenne psychiatry department; 2 Outpatient department, Razi Hospital, Tunis, Tunisia

## Abstract

**Introduction:**

Metabolic syndrome (MetS) is common in psychiatric patients and can be amplified by antipsychotic treatment. Beyond medication and lifestyle factors, studies suggest possible sex differences in vulnerability to MetS.

**Objectives:**

This study aimed to determine the prevalence of MetS in antipsychotic-treated psychiatric patients, stratified by sex, examining links with lifestyle and medication.

**Methods:**

A cross-sectional descriptive and analytical study, conducted between January and June 2025, at the Avicenne Psychiatry Department of Razi Hospital. Data were collected using a file and two arabic translated questionnaires:

The International Physical Activity Questionnaire (IPAQ): categorizing physical activity into: inactive, minimally active and HEPA-active.

The Mediterranean Diet Adherence Screener (MEDAS): with two categories: moderate to weak adherence ≤7; good to very good adherence ≥8.

MetS was defined according to NCEP ATP III criteria.

**Results:**

Forty-five patients were included with 67% being male. The mean age was 36.7 [19-62] years for men and 42.5 [23–60] years for women.

Among men, most participants (87%) were single, 87% were smokers and 10% consumed alcohol. While 83% had no underlying medical conditions, psychiatric diagnoses included schizophrenia (50%), bipolar I disorder (43%) and schizoaffective disorder (7%). Most (67%) were on atypical antipsychotics and 27% were on concurrent treatment with both typical and atypical agents. The median duration of antipsychotic treatment was 84 months.

Regarding physical activity, 37% were inactive, 40% were minimally active and 23% were HEPA active. Forty-three percent had good adherence to the mediterranean diet.

Among women, 47% were married, 40% were smokers and 7% consumed alcohol. Four women were menopausal.

No medical history was found in 53% of cases. Psychiatric diagnoses included schizophrenia (20%), bipolar disorder (I and II) (30%), schizoaffective disorder (20%) and other mental illnesses. While 80% were on atypical antipsychotics, 7% were on concurrent treatment with both typical and atypical agents. The median duration of antipsychotic treatment was 42 months.

In terms of physical activity, 47% were inactive and 53% were HEPA active. Thirty-three percent had good adherence to the mediterranean diet.

The overall prevalence of MetS was 33%. Prevalence was 27% in men and 47% in women (OR = 2.40).

There was no statistically significant difference in MetS prevalence between men and women (p = 0.18).

**Conclusions:**

This study indicates a higher prevalence of MetS among antipsychotic-treated Tunisian women, though not statistically significant, suggesting possible sex-specific vulnerability. These results call for further research to clarify determinants of MetS and guide targeted prevention.

**Disclosure of Interest:**

None Declared

